# The Acute Antiallodynic Effect of Tolperisone in Rat Neuropathic Pain and Evaluation of Its Mechanism of Action

**DOI:** 10.3390/ijms23179564

**Published:** 2022-08-24

**Authors:** Péter P. Lakatos, Dávid Árpád Karádi, Anna Rita Galambos, Nariman Essmat, Kornél Király, Rudolf Laufer, Orsolya Geda, Zoltán S. Zádori, Tamás Tábi, Mahmoud Al-Khrasani, Éva Szökő

**Affiliations:** 1 Department of Pharmacodynamics, Semmelweis University, 4 Nagyvárad tér, H-1089 Budapest, Hungary; 2 Department of Pharmacology and Pharmacotherapy, Semmelweis University, 4 Nagyvárad tér, H-1089 Budapest, Hungary

**Keywords:** neuropathic pain, tolperisone, pregabalin, CSF glutamate content, synaptosome, neuronal glutamate release

## Abstract

Current treatment approaches to manage neuropathic pain have a slow onset and their use is largely hampered by side-effects, thus there is a significant need for finding new medications. Tolperisone, a centrally acting muscle relaxant with a favorable side effect profile, has been reported to affect ion channels, which are targets for current first-line medications in neuropathic pain. Our aim was to explore its antinociceptive potency in rats developing neuropathic pain evoked by partial sciatic nerve ligation and the mechanisms involved. Acute oral tolperisone restores both the decreased paw pressure threshold and the elevated glutamate level in cerebrospinal fluid in neuropathic rats. These effects were comparable to those of pregabalin, a first-line medication in neuropathy. Tolperisone also inhibits release of glutamate from rat brain synaptosomes primarily by blockade of voltage-dependent sodium channels, although inhibition of calcium channels may also be involved at higher concentrations. However, pregabalin fails to affect glutamate release under our present conditions, indicating a different mechanism of action. These results lay the foundation of the avenue for repurposing tolperisone as an analgesic drug to relieve neuropathic pain.

## 1. Introduction

Despite the progress in the discovery of different analgesics, neuropathic pain treatment has not been successfully achieved. The International Association for the Study of Pain defines neuropathic pain as, “Pain caused by a lesion or disease of the somatosensory nervous system”. Damage to sensory nerves generates central sensitization, which manifests as developing pain to non-noxious stimuli called allodynia. Central sensitization is a result of functional, chemical, and structural plasticity that affects the central nociceptive system (for review see: [1]). Several transmitters are involved in this process including glutamate, which is considered as a substantial contributor to the sensitization of excitatory spinal dorsal horn interneurons [2]. Reduction of the central inhibitory systems, alongside enhancement of the central excitatory systems simultaneously occurs in the dorsal spinal cord of animals with neuropathic pain (for review see: [2]). A notable increase in glutamatergic neurotransmission has been reported alongside an elevation of glutamate levels in rodent neuropathic pain models ([3]; for review see: [4]). In addition, upregulation of voltage-gated sodium channel subtype Nav1.7 and the α2δ subunit of calcium channels has also been linked to neuropathic pain ([5,6]; for review see: [7]). Neuronal glutamate release is extra-operated by Ca^2+^ influx through voltage-gated calcium channels (VGCCs.). With respect to pain, spinal Cav2.2-mediated Ca^2+^ influx initiates the release of glutamate, which is involved in forwarding pain signals to the brain. In addition to the ascending pain pathway, VGCCs have also been shown to be involved in descending analgesic pathways [8]. Furthermore, potassium channels, which play a crucial role in neuronal action potential by counterbalancing sodium influx, are in turn down-regulated [9]. All these changes, among others, can lead to increased excitability of nociceptive neurons (for review see: [7]). These data reflect an imbalance between the excitatory neurotransmitter glutamate and inhibitory transmitters under neuropathic pain circumstances. Drugs that restore the balance between spinal inhibitory and excitatory systems could be of clinical significance regarding neuropathic pain management. Indeed, drugs inhibiting—directly or indirectly—the enhanced glutamatergic system are principal components of the present pharmacological approach for neuropathic pain treatment [2,10].

Tolperisone is a centrally acting muscle relaxant that was first introduced by Gedeon Richter Plc, Hungary. Its efficacy and safety have been demonstrated by clinical trials for the treatment of post-stroke spasticity [11] and painful reflex muscle spasms [12]. Despite its widespread use, the exact mechanism of action of tolperisone is yet to be elucidated, though an inhibitory effect on both sodium and calcium channels has been proposed. Tolperisone was shown as being able to attenuate the sodium current in Xenopus oocytes and to inhibit voltage-gated sodium channel isoforms Nav1.6, Nav1.7, and Nav1.8 with a potency that is comparable to or even higher than that of lidocaine [13,14]. In addition to sodium channel inhibition, Novales-Li et al. observed its concentration-dependent suppressive effect on calcium currents measured on Achatina fulica neurons under voltage clamping [15].

The inhibitory effect of tolperisone on excitatory ion channels, especially on the voltage-gated sodium channel isoform Nav1.7, raises the possibility of its beneficial action in neuropathy, as an inhibition of hyperactive nociceptive pathways is a major target to relieve neuropathic pain (for review see: [16]). To the best of our knowledge, this effect of tolperisone has not yet been explored. According to current guidelines, both sodium and calcium current inhibitors, such as topical lidocaine and gabapentinoids, are cornerstones in the therapeutic management of neuropathic pain [2,17].

The present work was carried out to examine whether tolperisone could reduce neuropathic pain in rats induced by partial sciatic nerve ligation (pSNL) and modulate spinal cord glutamatergic systems. Furthermore, its effect on glutamate release was also explored in various experimental settings to better understand its mechanism of action.

## 2. Results

### 2.1. Tolperisone Produces Antiallodynic Effect in Neuropathic Pain Evoked by pSNL

**Figure 1:**
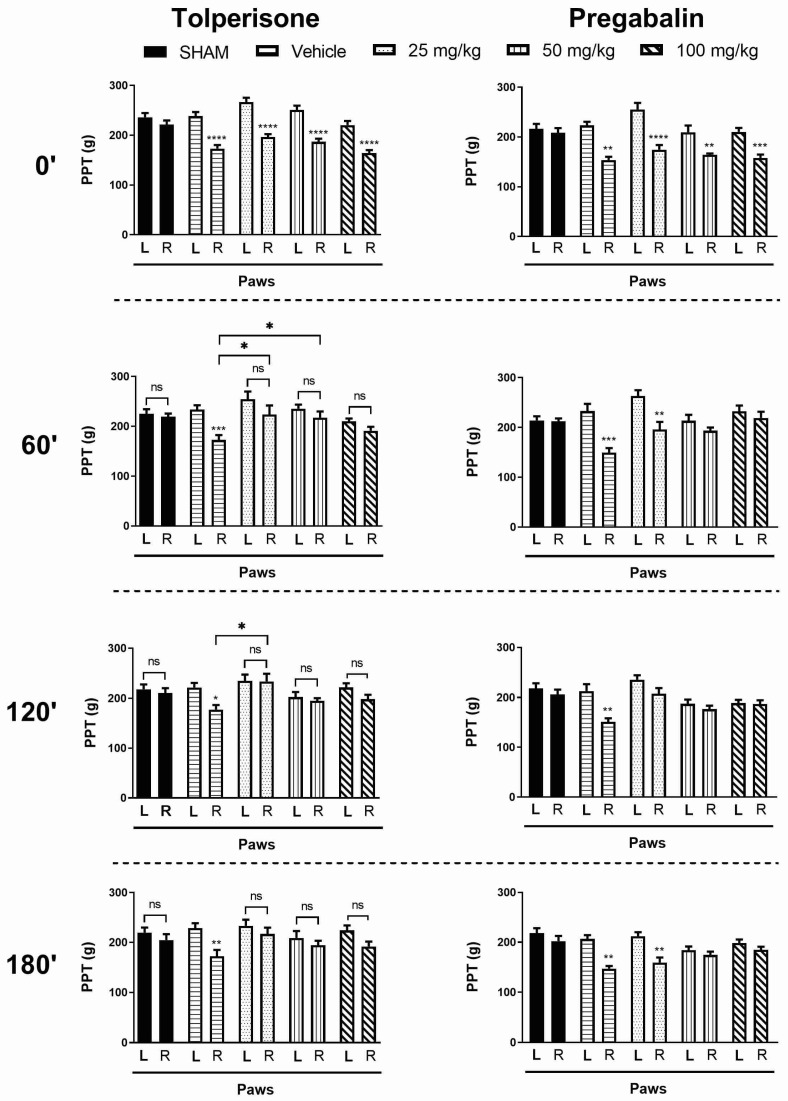
The antiallodynic effect of tolperisone (**left** panels) and pregabalin (**right** panels) following acute per os treatment (25, 50, 100 mg/kg). Graphs show the means of PPT *±* S.E.M. in grams of animals’ left (healthy, L) and right (operated, R) paws before (baseline) and after treatment (60 min; 120 min; 180 min) with either tolperisone or pregabalin. Asterisks mark the significant differences compared to left (healthy) paws or vehicle treated group (one-way ANOVA, F(9, 104) = 16.17 (tolperisone 0′), F(9, 104) = 5.182 (tolperisone 60′), F(9, 92) = 3.354 (tolperisone 120′), F(9, 92) = 3.028 (tolperisone 180′), F(9, 82) = 11.15 (pregabalin 0′), F(9, 82) = 5.955 (pregabalin 60′), F(9, 82) = 5.464 (pregabalin 120′), F(9, 82) = 6.234 (pregabalin 180′), Newman–Keuls post-hoc test; ****: *p* < 0.0001; ***: *p* < 0.001; **: *p* < 0.01; *: *p* < 0.05; ns: non-significant). In each treatment group 6–16 animals were used.

Figure 1 shows the antiallodynic effect of oral tolperisone compared to pregabalin in pSNL-induced mechanical allodynia indicated by a decrease in rat paw pressure thresholds (PPTs) measured by a Randall–Selitto test. Mechanical allodynia is a characteristic symptom of neuropathic pain [18]. Tolperisone in oral doses of 25, 50, and 100 mg/kg restored the developed mechanical allodynia in the tested time points namely 60, 120, and 180 min after treatment (Figure 1). Pregabalin as a positive control only at higher tested doses (50 and 100 mg/kg) produced consistent antiallodynic effect that remained unchanged until 180 min (Figure 1). On the other hand, vehicle-treated rats displayed mechanical allodynia by a significant decrease in the PPT of operated paws versus the non-operated ones (Figure 1).

### 2.2. Tolperisone and Pregabalin Treatments Reduce Elevated Cerebrospinal Fluid (CSF) Glutamate Level in Neuropathic Rats

**Figure 2:**
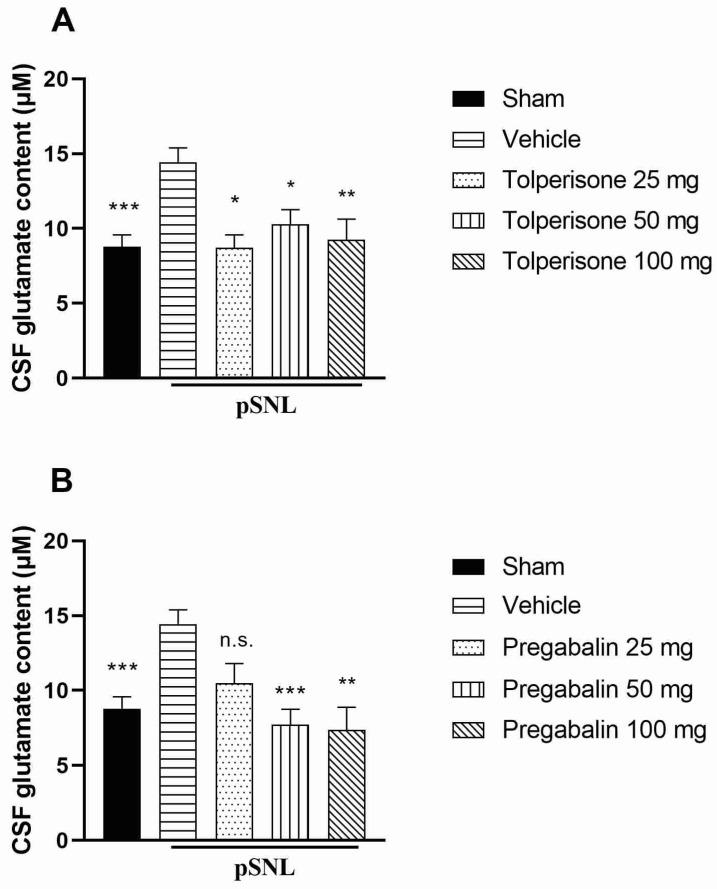
Glutamate content in CSF obtained from pSNL rats 14 days after surgery. Rats were treated with 25, 50, and 100 mg/kg dose of tolperisone (**A**) or pregabalin (**B**) per os or vehicle (**A**,**B**), and CSF samples were taken 3 h after treatment. Columns represent the mean of amino acid content *±* S.E.M. in µM in the indicated groups. Asterisks mark the significant differences compared to vehicle treated group (one-way ANOVA, F(4, 54) = 6.774 (**A**), F(4, 50) = 8.478 (**B**), Tukey post-hoc test; ***: *p* < 0.001; **: *p* < 0.01; *: *p* < 0.05; ns: non-significant). In each treatment group 6–18 animals were used.

A significant elevation of the CSF glutamate level was found in animals that developed mechanical allodynia [10] (Figure 2). All examined doses (25, 50, 100 mg/kg) of tolperisone normalized the neuropathy-induced elevation of CSF glutamate level measured 180 min after a single, oral treatment (Figure 2A). Pregabalin similarly restored the glutamate level of CSF of allodynic animals (Figure 2B).

### 2.3. Tolperisone Inhibits 4-Aminopyridine-Induced Glutamate Release from Rat Synaptosomes

**Figure 3:**
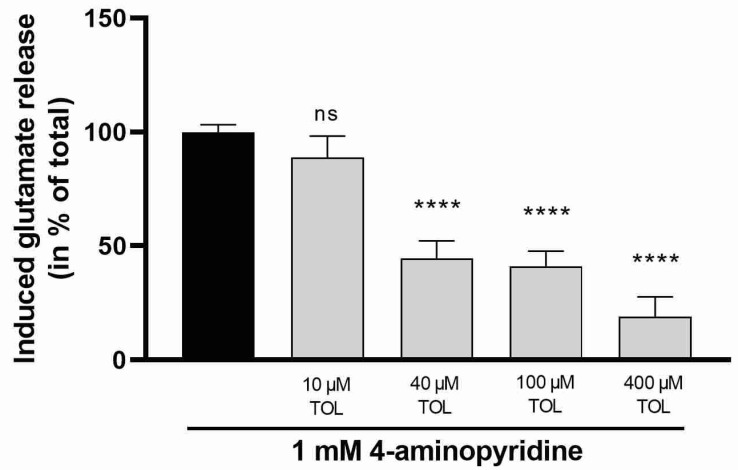
Effect of tolperisone on glutamate release from rat brain synaptosomes evoked by 1 mM 4-aminopyridine. Tolperisone was administered as a pretreatment 20 min prior to stimulation. Concentration of released glutamate was measured 6 min after stimulation. All data points were normalized using the unstimulated, baseline release and presented as % of the stimulated glutamate release in the absence of test compounds (black bar). All columns represent mean of glutamate release *±* S.E.M. in % in the indicated groups. Asterisks mark the significant differences compared to stimulated glutamate release in the absence of test compounds (one-way ANOVA, F(4, 44) = 33.63, Tukey post-hoc test; ****: *p* < 0.0001; ns: non-significant). In each treatment group 4–30 parallel experiments were used.

The effect of tolperisone on depolarization-evoked glutamate release from rat brain synaptosomes was examined to better understand its probable mode of action. Depolarization and subsequent neurotransmitter release were induced by 4-aminopyridine, a K^+^-channel inhibitor [19]. Transmitter release evoked by 4-aminopyridine is dependent on the activation of both sodium and calcium channels [20]. Tolperisone (40, 100, 400 µM) inhibited the 4-aminopyridine-induced glutamate release in a concentration-dependent manner (Figure 3).

**Figure 4:**
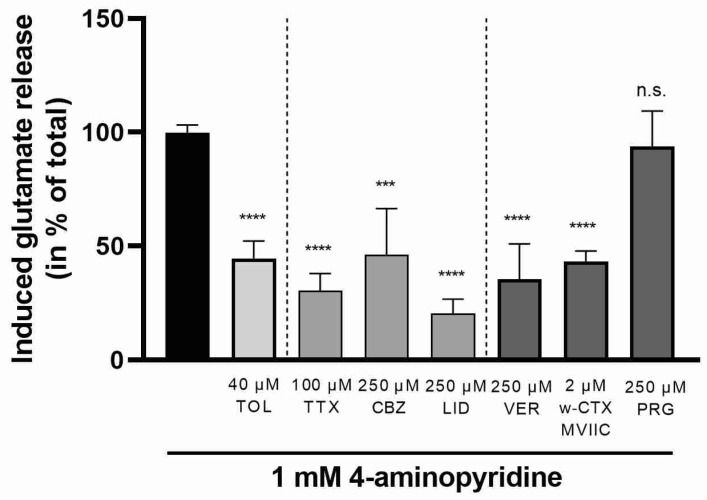
Effect of tolperisone, sodium channel blockers (TTX—tetrodotoxin; CBZ—carbamazepine; LID—lidocaine), and calcium channel blockers (VER—verapamil; ω-CTX—ω-conotoxin; PRG—pregabalin) on glutamate release from rat brain synaptosomes evoked by 1 mM 4-aminopyridine. Tolperisone and channel blockers were administered as a pretreatment 20 min prior to stimulation. Concentration of released glutamate was measured 6 min after stimulation. All data points were normalized using the unstimulated, baseline release and presented as % of the stimulated glutamate release in the absence of test compounds (black bar). All columns represent mean of glutamate release *±* S.E.M. in % in the indicated groups. Asterisks mark the significant differences compared to stimulated glutamate release in the absence of test compounds (one-way ANOVA, F(7, 50) = 16.83, Tukey post-hoc test; ****: *p* < 0.0001; ***: *p* < 0.001; ns: non-significant). In each treatment group 4–30 parallel experiments were used.

The effect of tolperisone was compared to those of known sodium and calcium channel inhibitors. Similarly to the action of tolperisone, a reduction in 4-aminopyridine induced glutamate release was observed in the case of voltage-gated sodium channel inhibitors tetrodotoxin, carbamazepine, lidocaine, and the selective N- and P-type voltage-gated calcium channel inhibitor, ω-conotoxin MVIIC [21], as well as verapamil, a non-selective calcium channel blocker in high concentrations [22] (Figure 4). Pregabalin, however, a selective inhibitor of calcium channels containing α2δ-subunits [23] was ineffective (Figure 4).

### 2.4. Tolperisone Inhibits Potassium-Induced Glutamate Release from Rat Synaptosomes

**Figure 5:**
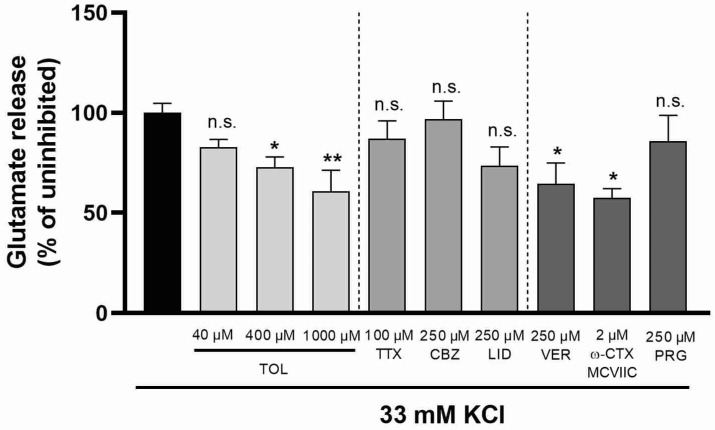
Effect of tolperisone, sodium channel blockers (TTX—tetrodotoxin; CBZ—carbamazepine; LID—lidocaine), and calcium channel blockers (VER—verapamil; ω-CTX—ω-conotoxin; PRG—pregabalin) on glutamate release from rat brain synaptosomes evoked by 33 mM potassium chloride. Tolperisone and channel blockers were administered as a pretreatment 20 min prior to stimulation. Concentration of released glutamate was measured 6 min after stimulation. All data points were normalized using the unstimulated, baseline release and presented as % of the stimulated glutamate release in the absence of test compounds (black bar). All columns represent mean of glutamate release *±* S.E.M. in % in the indicated groups. Asterisks mark the significant differences compared to stimulated glutamate release in the absence of test compounds (one-way ANOVA, F(7, 40) = 3.599, Tukey post-hoc test; *: *p* < 0.05; **: *p* < 0.01; ns: non-significant). In each treatment group 4–19 parallel experiments were used.

The action of tolperisone on high potassium concentration-induced (33 mM) glutamate release was also studied. A high extracellular concentration of potassium is known to elicit depolarization-evoked neurotransmitter release that is largely independent of voltage gated sodium channels by directly activating calcium currents [24]. As it was expected, sodium channel inhibitors tetrodotoxin, carbamazepine, and lidocaine all failed to inhibit potassium-evoked glutamate release (Figure 5), while the calcium channel blockers, verapamil and ω-conotoxin MVIIC, both caused a marked decrease in neurotransmitter release (Figure 5). In these experiments, tolperisone also decreased glutamate release but only in a high concentration (Figure 5), while 4-aminopyridine-evoked transmitter release was significantly inhibited by 40 µM tolperisone, a one order of magnitude higher concentration of the compound was necessary to significantly reduce potassium-induced glutamate release, indicating that it is a more potent inhibitor of sodium than calcium channels. Pregabalin was ineffective in decreasing glutamate release in this model, too (Figure 5).

## 3. Discussion

Despite the large arsenal of drug and non-drug approaches used in the management of different pain entities, patients with neuropathic pain do not respond fully to, or tolerate, the current pain medications [2,25]. In addition, clinical data indicate that current treatments are not equally effective in all types of neuropathy or in all patients, and the onset of their maximum effect is rather slow [25]. For instance, gabapentinoids and noradrenaline/serotonin reuptake inhibitor antidepressants are considered as first line drugs in the management of neuropathic pain, however they are not able to show equianalgesia in the different neuropathic pain types [26]. Therefore, neuropathic pain is in dire need of effective treatments with a fast onset and substantial analgesic effect in its various types. Repurposing of already approved medications is an attractive approach for drug research, rapidly providing effective therapies. Our present study raises the possibility that tolperisone might be used to manage neuropathic pain because its oral administration acutely ameliorated mechanical allodynia in rats with painful peripheral mononeuropathy evoked by pSNL [27]. As highlighted in the introduction, tolperisone is a drug used to reduce pathological overactivation of motor neurons yet hosting pharmacological profiles that can also inhibit hyperexcitability of sensory neuronal system, which typically contributes to the pathomechanism of neuropathic pain. In this regard, glutamate is considered as the major mediator of excitatory signals in the central nervous system (CNS) [28,29]. An elevated extracellular glutamate level is an important cause of increased activation of spinal dorsal horn neurons after nerve injury, thus impact of tolperisone on spinal glutamate transmission in rats developing mechanical allodynia was also investigated. CSF samples were thus obtained from neuropathic rats treated with tolperisone and assayed for glutamate level. The present results showed that CSF glutamate level was significantly increased in rats with neuropathy and this elevated concentration was restored by acute tolperisone treatment. Under chronic neuropathic pain condition, glutamate release from primary afferents or spinal interneurons, as well as an increase in glutamate level in CSF have been previously demonstrated [10,30,31]. Herein, we provided further evidence, which is consistent with previous results reported by our research group and others to support an increased CSF glutamate level after nerve ligation, which in turn can serve as an indicator of enhanced glutamatergic neurotransmission in neuropathy [10,32]. In the present work, we could show that oral tolperisone acutely relieve neuropathic pain. This effect was accompanied by restoration of the elevated CSF glutamate level further supporting the linkage between allodynia and an increased extracellular glutamate level. As a comparison, pregabalin, a first-line medication of neuropathic pain was applied as a positive control. Tolperisone showed similar antiallodynic activity compared to pregabalin following acute administration, suggesting it has considerable analgesic potential in mononeuropathy after nerve injury. Furthermore, both drugs reduce the elevated CSF glutamate level, suggesting amelioration of the facilitated spinal glutamatergic neurotransmission after nerve damage. Neither drug decreased the glutamate level below baseline, indicating that they selectively inhibit the pathological function of damaged neurons (for review see: [7]), likely by inhibiting the increased excitability due to altered expression of ion channels. 

According to the present data tolperisone might be a promising new tool in the treatment of neuropathic pain in doses that were already proven safe in the therapy of muscle spasticity. It is worth noting that acute oral tolperisone at a dose of 150 mg/kg avoided producing motor dysfunction (Supplementary Figure S1). On the other hand, previously significant motor dysfunction was reported following oral pregabalin treatment even in lower doses [33,34]. Our present results thus brought evidence of the effectiveness of tolperisone in neuropathic pain; however, further studies are necessary to fully characterize its antinociceptive effect in various chronic pain entities associated with neuropathy.

To better understand the role of various ion channels involved in the effect of tolperisone, in vitro experiments were performed on rat brain synaptosomes. Synaptosomes are extensively used for studying neurotransmission and its modulation by pharmacological tools. They contain membrane vesicles formed from the synaptic terminal and used to model the mechanism of neurotransmitter release [35]. As our ex vivo experiments indicated that tolperisone is able to reduce extracellular glutamate level in CNS, its direct effect on glutamate release was also studied. Two different means of stimulation were used, allowing us to assess the involvement of sodium and/or calcium channel inhibition in the effect of tolperisone. A selective A-type potassium channel inhibitor, 4-aminopyridine causes synaptic depolarization and induces tetrodotoxin-sensitive and calcium channel-dependent glutamate release. It evokes repeated action potentials closely resembling physiological neuronal activities [20]. In another set of experiments, depolarization was elicited by a high concentration of potassium that causes a significant shift in membrane potential that stabilizes at a less negative value and induces direct activation of voltage-gated calcium channels. During this type of stimulation non-desensitizing ion channels dominate [36] and the release mechanism cannot be inhibited by sodium channel blockade [24]. Comparing the effect of tolperisone on the release induced by 4-aminopyridine and high potassium concentration can thus distinguish between the involvement of sodium and calcium channel inhibition.

We have demonstrated for the first time that tolperisone concentration-dependently inhibits 4-aminopyridine-induced glutamate release, likely playing a prominent role in both its muscle relaxant and analgesic effect. A significant reduction in glutamate release was seen in the presence of as low as 40 µM of tolperisone. Its effect was comparable to those of tetrodotoxin and other sodium channel blockers. A higher concentration of 400 µM tolperisone, however, had a tendency for more effective inhibition of glutamate release that raises the possibility of at least two distinct mechanisms participating in its action within the examined concentration range. This difference may be explained by the capability of tolperisone to inhibit both tetrodotoxin-resistant and sensitive sodium channel subtypes [14] similarly to lidocaine [37] or the direct blockade of calcium channels. When high potassium concentration was used to elicit glutamate release, the effect of tolperisone was much less pronounced and only its high concentrations showed some inhibitory activity. In the 40 µM concentration that had a significant effect in case of 4-aminopyridine stimulation, it failed to inhibit glutamate release similarly to all of the examined sodium channel inhibitors. However, 400 and 1000 µM of tolperisone caused a significant decrease in potassium induced glutamate release similarly to a high concentration of verapamil, a non-selective inhibitor of high-threshold calcium channels, and ω-conotoxin MVIIC, a selective N- and P-type voltage-gated calcium channel inhibitor. Our present results suggest that tolperisone acts as a relatively selective sodium channel inhibitor that is capable of reducing glutamate release and normalizing the increased excitatory transmission in neuropathy induced by pSNL, although in a higher concentration it may directly inhibit calcium channels involved in transmitter release, and this mechanism might also contribute to its in vivo antiallodynic effect.

Interestingly, despite having a similar antiallodynic effect to tolperisone, pregabalin failed to inhibit glutamate release elicited by either 4-aminopyridine or high-concentration potassium. Pregabalin is regarded as a selective modulator of α2δ subunit-containing high threshold calcium channels in the CNS that participate in synaptic transmission. In a similar in vitro study, Kammerer et al. reported a comparable result and as an explanation proposed that pregabalin may act by blocking only postsynaptic calcium channels without affecting glutamate release [38]. Furthermore, Bauer et al. reported that pregabalin normalizes presynaptic α2δ subunit expression in pSNL rats by impairing its presynaptic trafficking. They proposed that the inhibitory effect of pregabalin on calcium channel expression rather than a direct blockade is responsible for its antinociceptive property [39]. Their hypothesis is supported by our present results, as pregabalin is deemed effective for restoration of elevated CSF glutamate levels in neuropathic rats, while it is incapable of modulating glutamate release from rat brain synaptosomes prepared from untreated animals. The compound might thus show selectivity to the pathologically upregulated calcium influx after nerve injury. Although tolperisone and pregabalin showed comparable antiallodynic effects in vivo, our results also indicate a considerable difference in their mechanism of action. The complementary mechanisms of the two compounds may be advantageous as they can cover a broader spectrum of pathological alterations seen in neuropathy and even their combination might be considered in some complicated cases. Further future studies would help to better characterize the discovered antinociceptive activity of tolperisone and open a promising way for its repurposing for the treatment of neuropathic pain.

Some limitations of the present studies should be noted. To reduce the number of sacrificed experimental animals, neuropathic pain was detected by a single method. Although the Randall–Selitto test is a widely accepted and robust way for detection of mechanical allodynia, another test could have further confirmed the antinociceptive effect of the tested drugs. Glutamate release experiments were carried out on synaptosomes isolated from healthy rats. Pathological alterations developing after nerve injury may have an impact on the observed effects of examined compounds.

## 4. Materials and Methods

### 4.1. Animals

Male Wistar rats were obtained from Toxi-Coop Zrt. (Budapest, Hungary). All animals were kept in standard cages in numbers of 4 or 5 animals/cage, depending on their weight, in a room of 20 ± 2 °C temperature, with 12 h/12 h light/dark cycle and with water and standard food available ad libitum. A total of 103 animals were used for Randall–Selitto tests, 9 for synaptosome preparation, and 80 for capillary electrophoresis analysis.

### 4.2. Chemicals

The following were obtained from Sigma-Aldrich (St. Louis, MO, USA): l-Glutamate, l-cysteic acid, 4-(2-hydroxyethyl)-1-piperazineethanesulfonic acid (HEPES), acetonitrile, boric acid, phenobarbital, sucrose, calcium chloride, potassium chloride, magnesium chloride, DMSO, verapamil hydrochloride, carbamazepine, and 4-aminopyridine. From the Tokyo Chemical Industry (Tokyo, Japan), the following was purchased: 4-fluoro-7-nitrobenzofurazan (NBD-F). The following were provided by Cyclolab Ltd (Budapest, Hungary): β-cyclodextrin and 6-monodeoxy-6-mono(3-hydroxy) propylamino -β-cyclodextrin hydrochloride. The following were obtained from Tocris Bioscience (Bristol, UK): dl-threo-β-benzyloxyaspartic acid (dl-TBOA), tetrodotoxin, and ω-conotoxin MVIIC. The following were purchased from Reanal (Hungary, Budapest): sodium chloride and D-glucose. Fetal bovine serum was obtained from Biosera (Nuaille, France). Lidocaine hydrochloride was provided by Egis Pharmaceutical Plc. (Budapest, Hungary). Tolperisone and pregabalin were received as kind gifts from Meditop Pharmaceuticals Ltd. (Budapest, Hungary). Ultrapure water from MilliQ Direct 8 water purification system (Merck, Darmstadt, Germany) was used for all experiments. All compounds were stored and handled as described in the product information sheets.

### 4.3. Experimental Protocols of the Animal Study

The applied experimental protocols were based on our previous publication [10]. Briefly, baseline measurements were performed using Randall–Selitto tests (see Section 4.5) to determine pain thresholds, followed by pSNL operations (see Section 4.4) On the 14th day after operation, mechano-hyperalgesia was assessed using Randall–Selitto tests. Afterward, compounds or vehicles were administered and mechano-hyperalgesia was measured again 60, 120, and 180 min after treatments in order to examine the acute effects of test compounds.

### 4.4. Partial Sciatic Nerve Ligation (pSNL)

For the induction of mononeuropathic pain in rats of 100–150 g, pSNL was applied, based on the Seltzer method [27,40]. In short, rats were anesthetized with 60 mg/kg intraperitoneal pentobarbital (2.5 mL/kg volume) and were put on a pillow of 30 °C. Under aseptic conditions the sciatic nerve of the right hind paw was carefully exposed without any muscle damage at the thigh-high level. Then, the nerve was tightly ligated with an 8-0 silicon-treated silk suture in a way that the dorsal 1/3–1/2 of the nerve thickness was trapped in the ligature. The wound was closed with 2 stiches. Sham-operated rats (with the nerve exposed without ligation) were used as controls.

### 4.5. Assessment of Mechanical Allodynia

Mechano-hyperalgesia (a major symptom of neuropathic pain) was assessed by paw pressure algesiometry (modified Randall–Selitto test; Ugo Basile, Comerio, Italy), as previously described [41,42,43]. PPTs were measured in grams after 5 min of habituation in the cage in the case of each measurement. PPT was measured three times on each paw and the average of the measurements was used for further analyses. For each animal, a minimum 20% decrease in the average PPT value of the operated (right) paw compared to the unoperated (left) paw was considered as development of mechanical allodynia. Sham-operated animals were used as controls.

### 4.6. Treatment of Animals

The effects of tolperisone and pregabalin (both at 25, 50, and 100 mg/kg) were investigated, after a single dose oral treatment on day 14 after pSNL. Drugs were dissolved in purificated water and administered via an orogastric gavage in a volume of 5 mL/kg. In each treatment 3–7 animals per group were used.

### 4.7. Capillary Electrophoresis Analysis of CSF Glutamate Content

Glutamate content of CSF samples was measured using a capillary electrophoresis-laser induced fluorescence detection method developed in our laboratory [44] with some modifications. Neuropathic and sham-operated rats were sacrificed 14 days after pSNL operation. CSF samples were obtained by cisterna magna puncture and centrifuged at 2000× *g*, 4 °C for 10 min. The samples were then deproteinized by mixing with 2 volumes of cold acetonitrile and centrifuged at 20,000× *g* for 10 min at 4 °C. Supernatants were subjected to derivatization with NBD-F (1 mg/mL final concentration) in 20 mM borate buffer pH 8.5 for 20 min at 65 °C. One µM l-cysteic acid was used as an internal standard. Derivatized samples were analyzed by a P/ACE MDQ Plus capillary electrophoresis system coupled with laser induced fluorescence detector set to 488 and 520 nm excitation and emission wavelengths, respectively (SCIEX, Framingham, MA, USA). Separations were carried out in polyacrylamide coated fused silica capillaries (i.d.: 75 µm, effective/total length: 40/50 cm), using 50 mM HEPES buffer pH 7.0 containing 6 mM 6-monodeoxy-6-mono(3-hydroxy) propylamino-β-cyclodextrin at 15 °C by applying −27 kV constant voltage.

### 4.8. Glutamate Release from Synaptosomes

The effects of tolperisone on depolarization-evoked release of glutamate was measured on rat brain synaptosomes prepared using a modified method based on Modi et. al. [45]. Briefly, animals were sacrificed by decapitation and their brain was rapidly removed and homogenized in a medium containing 0.32 M sucrose and 4 mM HEPES (pH 7.4). The homogenate was then centrifuged (2 × 10 min, 1500× *g*, 4 °C) and the supernatants were collected and combined. Afterward, the supernatant was centrifuged (2 × 10 min, 20,000× *g*, 4 °C) and the resulting pellet was resuspended in a buffer containing 0.32 M sucrose and 4 mM HEPES with 10% dimethyl sulfoxide (DMSO) and 10% fetal bovine serum and then stored at −80 °C until further use.

On the day of experiments, synaptosomal suspensions were thawed, centrifuged (10 min, 20,000× *g*, 4 °C), and the pellet was resuspended in 10 mM HEPES buffer containing 130 mM NaCl, 5.4 mM KCl, 1.3 mM CaCl_2_, 0.9 mM MgCl_2_, and 5.5 mM glucose (pH 7.4). Synaptosomal suspensions corresponding to 10 mg of synaptosomes were centrifuged to an 8-well strip plate (15 min, 2500× *g*, 4 °C) and the supernatant was discarded. Synaptosomes were equilibrated for 2 × 10 min at 37 °C before stimulation in HEPES buffer containing 40 µM dl-TBOA, a competitive, non-transportable blocker of excitatory amino acid transporters [11] to inhibit the reuptake of released glutamate. In the experiments, test compounds were added during the equilibration periods as pretreatment. After equilibration, stimulation buffer containing either 1 mM 4-aminopyridine or 33 mM potassium chloride was used to elicit depolarization and subsequent glutamate release. Following stimulation, aliquots were taken at 6 min and stored at −20 °C until capillary electrophoresis analysis.

### 4.9. Capillary Electrophoresis Analysis of Glutamate Released from Synaptosomes

Released glutamate was determined using a capillary electrophoresis-laser induced fluorescence detection method developed in our laboratory [46]. Samples were subjected to derivatization as described in Section 4.7. Measurements were carried out in polyacrylamide coated fused silica capillaries (i.d.: 75 µm, effective/total length: 10/50 cm), using 100 mM borate buffer pH 8.5, containing 8 mM β-cyclodextrin at 25 °C, by applying 18 kV constant voltage.

### 4.10. Statistical Analysis

All data were presented as mean ± S.E.M. Data were analyzed by one-way ANOVA in case of all experiments. For a comparison of multiple groups, Newman–Keuls (Randall–Selitto) and Tukey (CSF glutamate concentration, synaptosomal glutamate release) post-hoc tests were used, respectively. In the case of Randall–Selitto tests and CSF glutamate content measurement vehicle-treated animals, while in the release experiments stimulated, not pre-treated groups were considered as the control, respectively. Differences were considered significant if *p* < 0.05. Data analysis was carried out by statistical software Prism 8.0 (GraphPad Software Inc., San Diego, CA, USA).

## 5. Conclusions

Oral tolperisone was proved to have an acute antiallodynic effect in pain due to peripheral neuropathy in rats. Its antinociceptive activity is comparable to that of pregabalin, an established first line medication in clinical practice. Restoration of the elevated CSF glutamate level in neuropathy by primarily modulating the sodium ion channels involved in glutamate release likely contributes to the found antiallodynic effect of tolperisone. 

## Data Availability

The data that support the findings of this study are available from the corresponding author upon reasonable request.

## Supplementary Materials

The following supporting information can be downloaded at: https://www.mdpi.com/article/10.3390/ijms23179564/s1.
