# Supplementary material for: The Acute Antiallodynic Effect of Tolperisone in Rat Neuropathic Pain and Evaluation of Its Mechanism of Action
Source: Int J Mol Sci. 2022 Aug 24;23(17):9564. doi: 10.3390/ijms23179564 (PMC9455595; doi:10.3390/ijms23179564)
Supplement: Supplementary file 1 [file ijms-23-09564-s001.zip › Supplementary Figure S1 Legend.pdf]

Supplementary Figure S1. The effect of oral administration of tolperisone (150 mg/kg) compared to saline-treatment. Columns represent the time latency of animals in sec ± *S.E.M.* at 60, 120 and 180 min post-treatment in rotarod test.

No significant differences were found between any groups (one-way ANOVA, $F(5, 24) = 1.059$, $P = 0.4073$). In each treatment group 4-5 animals were used.
