# Supplementary figures and images for: The Acute Antiallodynic Effect of Tolperisone in Rat Neuropathic Pain and Evaluation of Its Mechanism of Action
Source: Int J Mol Sci. 2022 Aug 24;23(17):9564. doi: 10.3390/ijms23179564 (PMC9455595; doi:10.3390/ijms23179564)

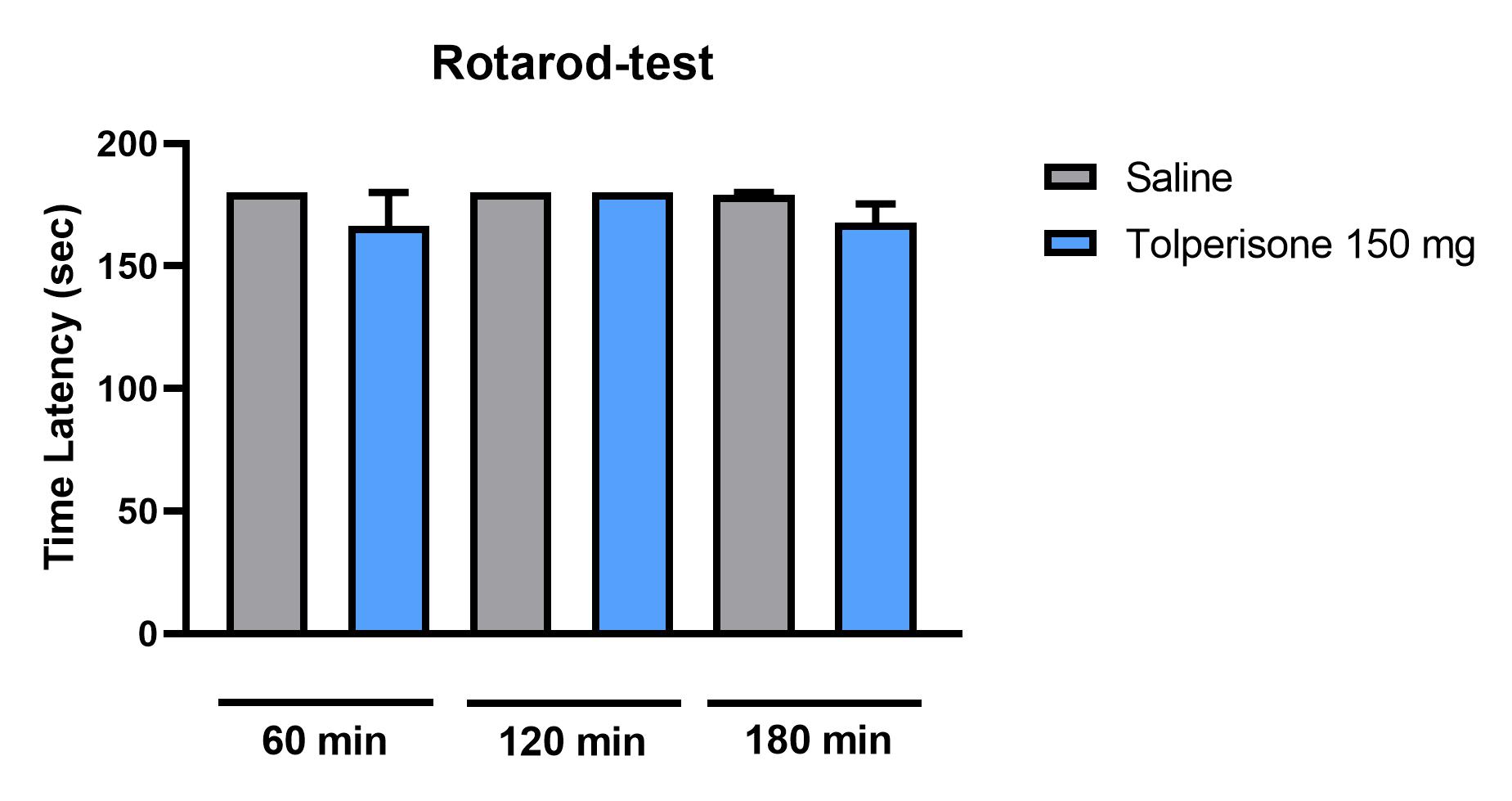

Supplement: Supplementary file 1 [file ijms-23-09564-s001.zip › Supplementary Figure S1.jpg]
